# Sequential nasal and mammary myeloid sarcoma with a 6-year latency: a case report and literature review

**DOI:** 10.3389/fmed.2026.1822818

**Published:** 2026-04-28

**Authors:** Jiaxin Yu, Chuanlin Hou, Feng Xu

**Affiliations:** 1 School of Medicine, Shaoxing University, Shaoxing, Zhejiang, China; 2 Department of General Surgery, Shaoxing People’s Hospital, Shaoxing, Zhejiang, China; 3 Department of Pathology, Shaoxing People’s Hospital, Shaoxing, Zhejiang, China; 4 The First Affiliated Hospital of Shaoxing University, Shaoxing, Zhejiang, China

**Keywords:** breast neoplasms, extramedullary relapse, myeloid sarcoma, nasal cavity, tumor dormancy

## Abstract

Myeloid sarcoma (MS) is a rare extramedullary manifestation of acute myeloid leukemia. While MS can involve various organs, isolated involvement of the nasal cavity or breast is uncommon, and the sequential involvement of both sites in a single patient is extremely rare. We present the case of a 68-year-old female who developed an MS relapse in the right breast 6 years after being treated for primary MS of the right nasal cavity. In 2018, she presented with a right nasal mass, initially suspected clinically to be a polyp. Histopathological and immunohistochemical (IHC) analyses confirmed MS. She achieved complete remission following localized radiotherapy and cytarabine-based systemic chemotherapy. After a six-year disease-free interval, she presented in 2025 with a painful right breast nodule. Initial imaging indicated a primary breast malignancy (BI-RADS 4B). However, postoperative IHC analysis, demonstrating myeloperoxidase (MPO) positivity, confirmed an extramedullary relapse of MS. The patient was subsequently treated with further chemotherapy and hypofractionated radiotherapy. This case highlights that extramedullary MS can perfectly mimic primary solid tumors both clinically and morphologically. It emphasizes the critical role of a comprehensive immunohistochemical panel, particularly the inclusion of myeloid-specific markers, in differentiating MS from poorly differentiated carcinomas. Such pathological vigilance is indispensable for avoiding catastrophic diagnostic pitfalls and preventing inappropriate surgical interventions in patients presenting with atypical extramedullary masses.

## Introduction

Extramedullary myeloid sarcoma (MS), historically known as granulocytic sarcoma, is a rare hematological malignancy characterized by the localized tumorous proliferation of immature myeloid cells outside the bone marrow (1, 2). MS can occur *de novo* as an isolated primary lesion, concurrently with acute myeloid leukemia (AML), or as a blast transformation in patients with underlying myeloproliferative neoplasms or myelodysplastic syndromes (3). Although relatively uncommon, isolated MS represents a clinically important phenomenon that frequently poses a significant diagnostic challenge in modern oncology (4). The pathogenesis of MS is multifactorial, involving complex interactions between leukemic blasts and the extramedullary microenvironment that facilitate tissue infiltration, immune evasion, and, occasionally, prolonged tumor dormancy (5).

While MS can theoretically involve any anatomical site, epidemiological studies and clinical observations indicate that the most frequently reported locations include the lymph nodes, skin, bones, central nervous system, and soft tissues (6). In contrast, isolated MS affecting the nasal cavity and the breast remains exceedingly rare and is highly prone to clinical misdiagnosis (7). For instance, breast involvement is observed in merely 0.12% of patients with AML, and specific data regarding nasal cavity MS are largely restricted to sporadic case reports (8). Furthermore, the metachronous (sequential) occurrence of MS in two distinct, highly atypical extramedullary sites, such as the nasal cavity followed by the breast, spanning a prolonged latency period, is exceptionally rare and sparsely documented in the literature (9).

Herein, we report an unusual clinical case of a patient who initially presented to our hospital with swelling and pain in the left submandibular region. No significant improvement was observed after oral antibiotic administration, and she subsequently developed progressive right nasal obstruction. She was ultimately diagnosed with isolated primary myeloid sarcoma of the right nasal cavity, following a six-year disease-free interval, experienced a metachronous extramedullary relapse in the right breast. This case aims to highlight the profound diagnostic challenges when extramedullary MS morphologically masquerades as a primary breast malignancy. By detailing this uncommon sequential presentation, we underscore the indispensable role of extended immunohistochemical screening in differentiating MS from poorly differentiated carcinomas and other small round cell tumors, thereby providing critical pathological insights to avoid clinical misdiagnosis.

## Case presentation

A 68-year-old female patient with a history of hypertension presented to our hospital with a one-day history of a newly discovered, painful mass in the right breast. Physical examination revealed a palpable, hard mass with ill-defined borders located at the outer lower quadrant of the right nipple, along with a 0.5 × 0.3 cm hard nodule on the overlying skin. No costovertebral angle tenderness was noted, and cardiopulmonary examination was unremarkable. A detailed review of her medical history revealed no family history of cancer and no major genetic cancer risk factors.

**Figure 1:**
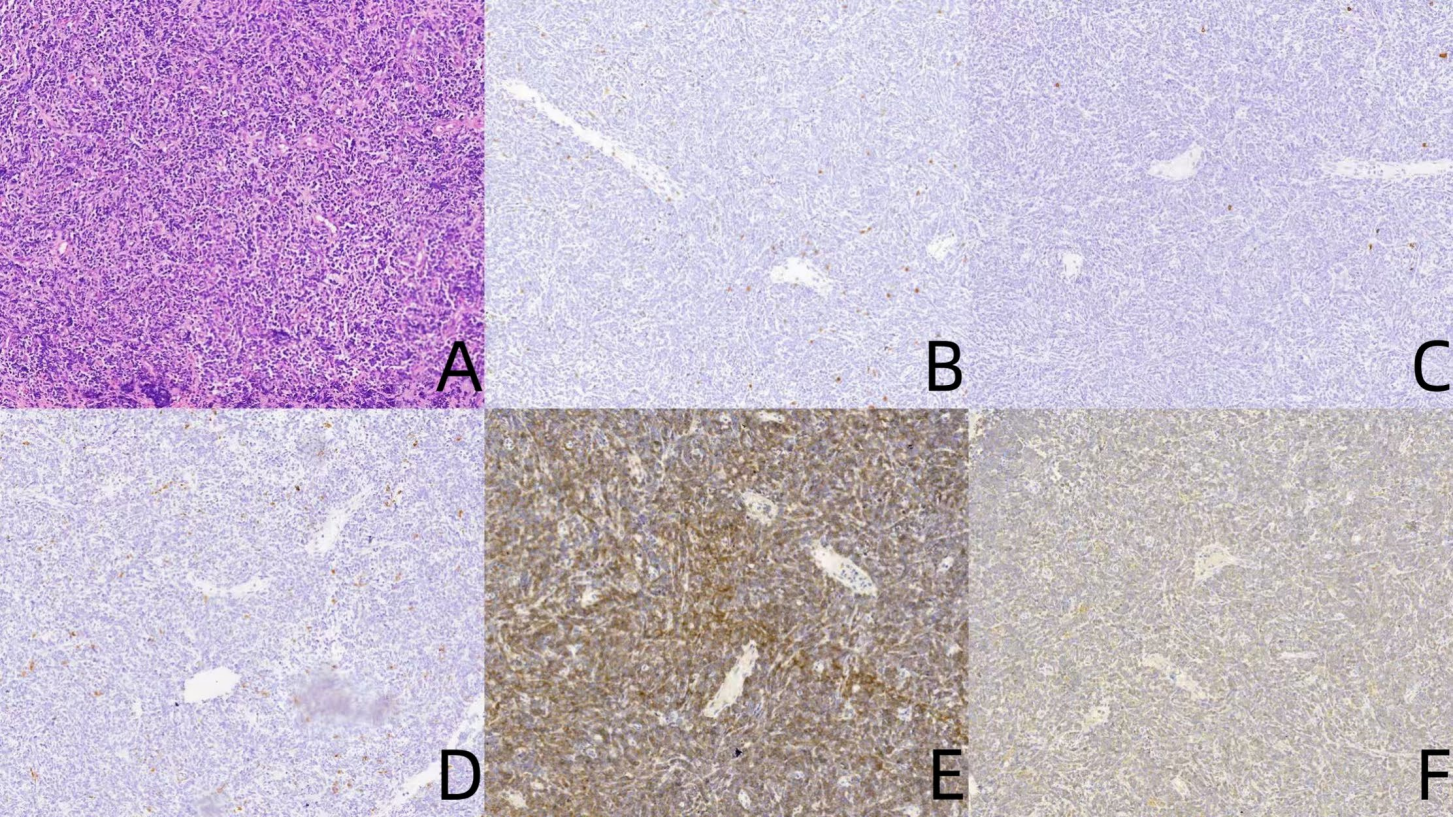
Pathological features of nasal cavity myeloid sarcoma. HE staining **(A)** demonstrates a diffuse proliferative lesion of lymphoid tissue with atypical spindle cells. Immunohistochemical staining shows negative expression for CD3 **(B)**, CD20 **(C)**, CD68 **(D)**, while positive expression for CD117 **(E)**, MPO **(F)**.

Approximately 6 years before the current admission, the patient initially sought medical attention in October 2018 for swelling and pain in the left submandibular lymph node. Despite oral antibiotic therapy, her symptoms persisted and progressed to right-sided nasal obstruction. Subsequent nasal endoscopy in February 2019 revealed a grayish-white mass in the right nasal cavity. A biopsy was performed, and histopathological examination demonstrated a diffuse proliferative lesion of lymphoid tissue with atypical spindle cells (Figure 1A). Comprehensive immunohistochemical (IHC) analysis revealed the following profile: the neoplastic cells were negative for CD3 (Figure 1B), CD20 (Figure 1C), CD68 (Figure 1D), and CD5, while demonstrating positivity for CD117 (Figure 1E), MPO (Figure 1F), CD43, CD99, and CD34 (vascular), with a Ki-67 proliferation index of approximately 60%. These findings supported the diagnosis of primary myeloid sarcoma (granulocytic sarcoma) of the nasal cavity.

At the time of the initial diagnosis, comprehensive clinical and pathological evaluations, including bone marrow aspiration and biopsy, revealed active hematopoiesis without evidence of acute myeloid leukemia (AML) involvement. Karyotype analysis showed 46, XX, and screening for AML prognostic gene mutations was negative. Following diagnosis confirmation, the patient received localized radiotherapy (15 fractions). She was subsequently transferred to our Hematology Department and initiated first-line systemic therapy, completing five cycles of the IA regimen (idarubicin 10 mg on days 1–3 and cytarabine 150 mg on days 1–7) between May and November 2019. Following the completion of treatment, regular outpatient follow-ups confirmed complete remission with no evidence of nasal cavity tumor recurrence.

**Figure 2:**
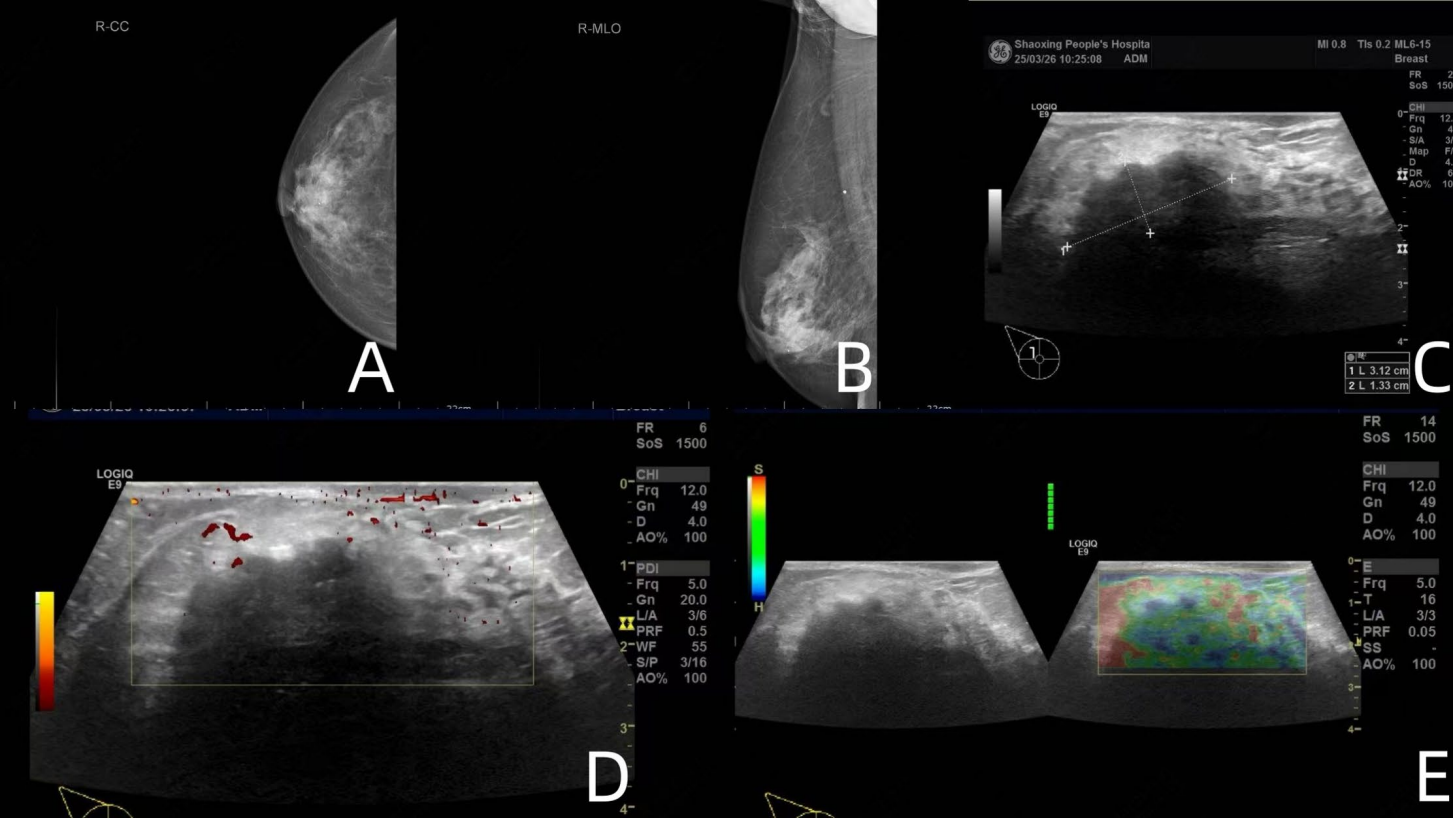
Mammography demonstrated a nodular hyperdense lesion measuring approximately 28 × 23 mm behind the right nipple, characterized by partially ill-defined borders and punctate calcification **(A,B)**. Breast ultrasonography further revealed a 31 × 13 × 29 mm hypoechoic nodule with irregular shape and peripheral blood flow signals **(C–E)**.

The patient remained disease-free for over 6 years. Returning to the current admission in March 2025, mammography demonstrated a nodular hyperdense lesion measuring approximately 28 × 23 mm behind the right nipple, characterized by partially ill-defined borders and punctate calcification, categorized as BI-RADS 4A (Figures 2A,B). Breast ultrasonography further revealed a 31 × 13 × 29 mm hypoechoic nodule with irregular shape and peripheral blood flow signals, categorized as BI-RADS 4B (Figures 2C–E).

**Figure 3:**
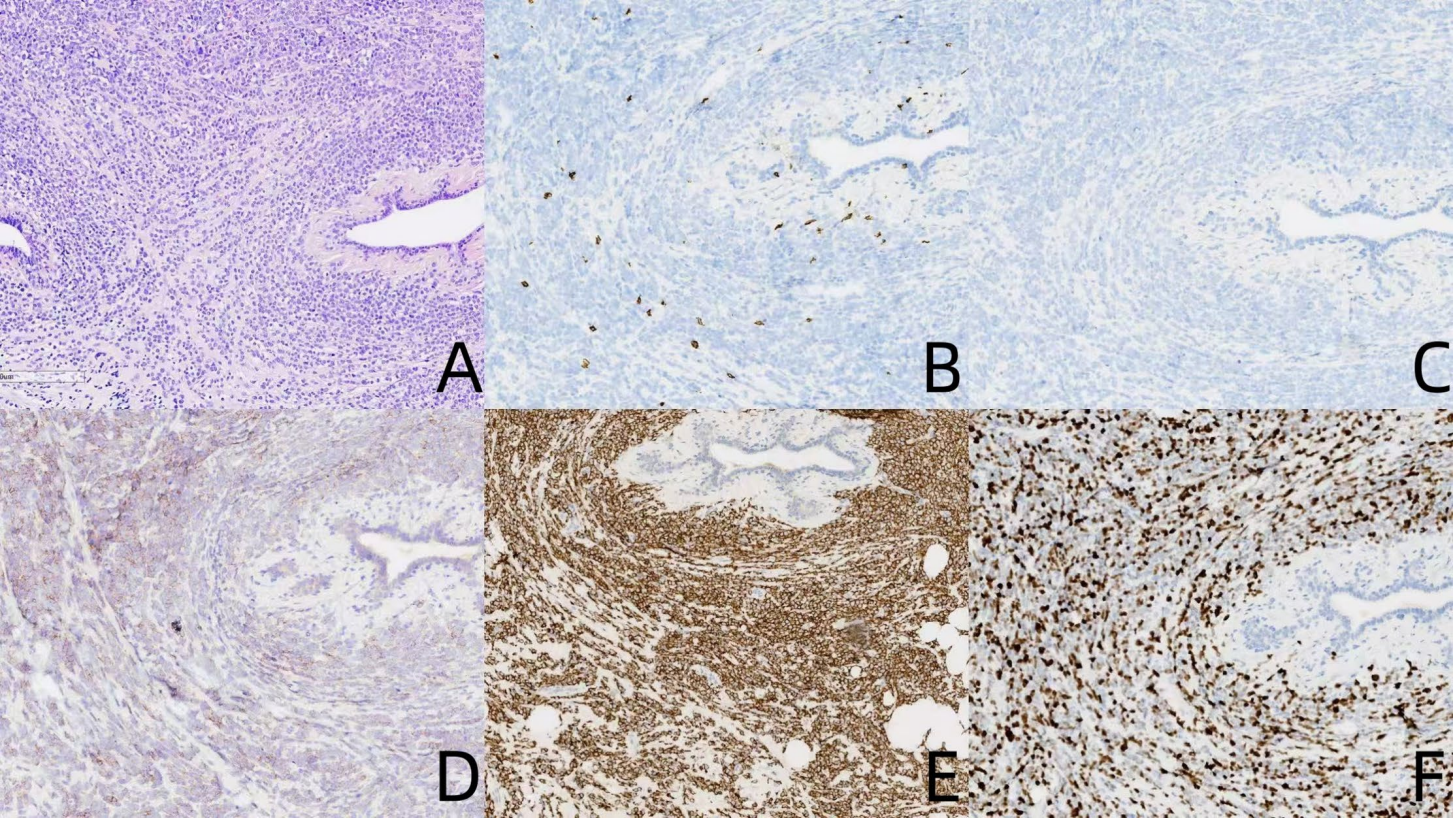
Pathological morphology of right breast myeloid sarcoma **(A)**. Immunohistochemical staining shows negative expression for CD3 **(B)**, CD20 **(C)**, while positive expression for CD43 **(D)**, MPO **(E)**, with a Ki-67 proliferation index of approximately 80% **(F)**.

After excluding surgical contraindications, the patient underwent bilateral breast lesion resection and excision of the right nipple skin mass. Intraoperative observation identified a 3.0 × 2.0 cm hard, unencapsulated mass in the right breast and a separate 1.0 × 0.5 cm soft, encapsulated mass in the left breast. Postoperative histopathological evaluation of the left breast mass indicated benign breast adenosis with fibroadenoma formation (Figure 3A). Conversely, the right breast mass and the overlying skin nodule revealed malignant tumor infiltration. IHC analysis of the right breast lesion demonstrated that the neoplastic cells were negative for CD3 (Figure 3B), CD20 (Figure 3C), CD15, and CD68, while remaining positive for CD43 (+) (Figure 3D), MPO (+) (Figure 3E), and BCL-2 (+), with weak positivity for CD117 and Ki-67 (+80%) (Figure 3F). Based on the patient’s clinical history and pathological characteristics, the final diagnosis was confirmed as an extramedullary relapse of myeloid sarcoma in the breast.

Following surgical intervention, the patient was transferred back to the Hematology Department. Re-evaluation of the bone marrow confirmed a small amount of hematopoietic tissue with moderately active proliferation, and flow cytometry remained negative for leukemic involvement. She subsequently underwent four additional cycles of cytarabine-based systemic chemotherapy (comprising cyclophosphamide and cytarabine) from April to August 2025. During chemotherapy, she developed transient myelosuppression and infection, which successfully resolved after symptomatic treatment. She then received hypofractionated radiotherapy targeting the right breast with a total dose of 2,400 cGy delivered in 8 fractions starting in October 2025. The patient’s condition remained stable postoperatively with a satisfactory clinical response, and she is currently undergoing regular outpatient follow-up.

## Discussion

The diagnosis of extramedullary myeloid sarcoma relies heavily on the identification of tumorous proliferation of immature myeloid cells outside the bone marrow. MS can manifest as a primary isolated lesion, occur concurrently with acute myeloid leukemia, or present as an extramedullary relapse following prior treatment (10). This classification underscores both the diverse biological behavior of the disease and the diagnostic complexity when bone marrow involvement is absent. The reported incidence of isolated MS is extremely low, and the breast is involved in merely 0.12% of patients with AML. Moreover, compared with single-site presentations, patients exhibiting metachronous (sequential) extramedullary relapses across distinct, highly atypical anatomical sites generally represent a much rarer clinical phenomenon with potentially more aggressive disease behavior.

In the present case, the sequential manifestation of MS in the nasal cavity and the breast fulfills the criteria for a metachronous extramedullary relapse characterized by profound tumor dormancy. First, both the initial nasal lesion and the subsequent breast tumor were histopathologically confirmed as MS without concurrent bone marrow leukemic transformation. Second, the interval between the diagnoses exceeded 6 years, a remarkable disease-free duration that strongly supports the classification of a dormant extramedullary relapse rather than continuous, treatment-resistant systemic progression.

From a pathological perspective, the diagnosis of MS in this case was supported by both morphological and immunophenotypic findings. Immunohistochemical (IHC) analysis of both the resected nasal and breast tumor specimens revealed diffuse positivity for myeloperoxidase (MPO) and CD117, alongside a high Ki-67 proliferation index. Histopathological examination demonstrated that the tumor cells were embedded within the host stroma, exhibiting hyperchromatic nuclei and atypical proliferation. Morphologically, MS in the breast is notoriously difficult to distinguish from poorly differentiated invasive breast carcinoma, lobular carcinoma, and other small round cell tumors, making it highly prone to catastrophic misdiagnosis. Without a high index of suspicion, pathologists might solely rely on routine epithelial markers, leading to an erroneous diagnosis of a primary solid tumor. Therefore, our case heavily underscores that incorporating myeloid-specific markers (such as MPO, CD117, and CD43) into the routine diagnostic algorithm is not merely an optional step, but an absolute necessity when evaluating atypical extramedullary lesions. Notably, prior studies have shown that the expression of myeloid-specific markers such as MPO is the gold standard for confirming granulocytic sarcoma, while the negativity of CD20 and CD3 effectively rules out B-cell and T-cell lymphomas (11). The precise immunophenotypic panel utilized in this case was instrumental in confirming the myeloid origin of the neoplastic cells across both temporal occurrences.

The pathophysiological mechanisms underlying prolonged tumor dormancy and subsequent extramedullary relapse in MS remain incompletely elucidated. Several factors have been implicated, including the existence of leukemic stem cells (LSCs), tumor immune evasion, and the protective role of specific tissue microenvironments. Following the initial cytarabine-based systemic chemotherapy and localized radiotherapy in 2019, the bulk of the tumor burden was eradicated. However, it is hypothesized that residual leukemic clones may have migrated to and survived within supportive extramedullary niches. These dormant cells can remain clinically silent for years, evading immune surveillance (12). The subsequent development of the breast lesion after 6 years suggests an eventual disruption of immune homeostasis or localized microenvironmental changes that triggered the reactivation and proliferation of these dormant clones. Accordingly, we present tumor dormancy and microenvironmental immune escape as plausible contributing factors driving this rare metachronous presentation. Nonetheless, further mechanistic studies and cytogenetic analyses, such as exploring mutations in FLT3-ITD or BCR-ABL1, are warranted to clarify the precise pathophysiological links underpinning this rare MS phenotype.

Due to its non-specific clinical manifestations and typically insidious onset, MS is frequently misinterpreted as a benign condition or a primary solid tumor. In the nasal cavity, MS patients may present with non-specific symptoms such as nasal obstruction, epistaxis, or facial swelling, which can easily be mistaken for common rhinitis or benign polyps, as initially suspected in our patient in 2018. By contrast, breast MS most commonly presents as a newly discovered breast mass. Mammography and ultrasonography often reveal hypoechoic or hyperdense nodules with irregular borders and internal blood flow, features that perfectly mimic primary invasive breast carcinoma (frequently categorized as BI-RADS 4 or 5) (13). The overlap of such subtle and heterogeneous symptoms highlights the diagnostic complexity of extramedullary MS and underscores the need for heightened clinical vigilance.

The clinical presentation of MS is nonspecific and typically presents as a breast mass. Nevertheless, the pain characteristics and associated symptoms of breast MS show distinct features that differ from primary breast carcinoma. The pain associated with MS is usually described as persistent distending pain, dull pain, or tenderness. In cases where the tumor invades the chest wall muscles, ribs, or compresses adjacent nerves, radiating pain or stabbing pain may occur. In contrast, early-stage primary breast cancer most commonly presents as a solitary, hard, ill-defined, and poorly mobile nodule. Patients are generally asymptomatic with no spontaneous pain, and the mass is often discovered incidentally during physical examination or self-palpation. Local dull pain or distending pain occurs only in a small proportion of patients with advanced breast cancer, secondary to tumor invasion into the chest wall, skin, or nerve compression. In conclusion, the key clinical feature of the current case was a painful breast mass, whereas early primary breast cancer is typically painless. Pronounced differences exist between these two entities in terms of pain presence, onset timing, and underlying pathological mechanisms. These distinct clinical features underscore the atypical clinical behavior of myeloid sarcoma involving the breast.

The management of MS requires close multidisciplinary collaboration among specialists in hematology, surgery, radiation oncology, and pathology. Effective treatment planning should comprehensively account for tumor location, patient performance status, and prior therapeutic exposures. Although there is no universally standardized treatment protocol exclusively for isolated MS, current systemic chemotherapy regimens for AML are widely considered the most appropriate first-line strategy (3). Regimens typically include cytarabine combined with anthracyclines (such as idarubicin). For localized control and the reduction of local recurrence risk, surgical resection followed by targeted radiotherapy is highly recommended (14). In the present case, the patient achieved a favorable clinical response through a combination of local surgical excision, cytarabine-based systemic chemotherapy, and hypofractionated radiotherapy.

Previous studies have demonstrated that the prognosis of MS is influenced by multiple variables, including the timing of appropriate intervention, cytogenetic abnormalities, and therapeutic response (15). Because MS has the potential to progress to systemic AML or relapse in unpredictable anatomical sites, achieving long-term remission requires aggressive initial systemic therapy coupled with meticulous follow-up. It was only through comprehensive histopathological re-evaluation of the breast mass that the secondary extramedullary relapse was accurately identified in this patient, preventing inappropriate treatment algorithms intended for primary breast cancer. Therefore, we underscore the critical importance of long-term follow-up in patients with a history of MS to monitor for both local recurrence and distant metachronous relapses (16). From a clinical surveillance standpoint, any newly developing mass in a patient with a history of MS, regardless of the anatomical site or the duration of the disease-free interval, must be approached with a high index of suspicion. Prompt biopsy and rigorous IHC evaluation utilizing myeloid-specific markers should be incorporated into the standard diagnostic workflow. A primary limitation of this study is its design as a single case report. Therefore, data collection from larger, multi-center cohorts is warranted to establish standardized therapeutic protocols and enable more robust prognostic assessments for patients experiencing late extramedullary relapses.

In conclusion, this report presents a rare and instructive case of metachronous MS involving the nasal cavity and the breast, separated by a six-year latency period. It provides valuable clinical insights that contribute to the existing literature on tumor dormancy and diagnostic pitfalls in MS. The underlying mechanisms responsible for this sequential extramedullary development remain incompletely elucidated and warrant further investigation to clarify the pathogenesis and optimize long-term management strategies.

## Data Availability

The original contributions presented in the study are included in the article/supplementary material, further inquiries can be directed to the corresponding author.
